# The first checklist of alien vascular plants of Kyrgyzstan, with new records and critical evaluation of earlier data. Contribution 1

**DOI:** 10.3897/BDJ.9.e75590

**Published:** 2021-11-09

**Authors:** Alexander N. Sennikov, Georgy A. Lazkov

**Affiliations:** 1 University of Helsinki, Helsinki, Finland University of Helsinki Helsinki Finland; 2 Komarov Botanical Institute, Saint-Petersburg, Russia Komarov Botanical Institute Saint-Petersburg Russia; 3 Institute of Biology, Bishkek, Kyrgyzstan Institute of Biology Bishkek Kyrgyzstan

**Keywords:** archaeophytes, Asteraceae, botanical gardens, Brassicaceae, *Bunias*, Central Asia, Compositae, *Erigeron*, established aliens, introduction, naturalisation, neophytes, non-native plants, plant invasions, war-time immigrants, *Xanthium*

## Abstract

**Background:**

National checklists of alien plants and detailed databases of non-native plant occurrences are required to study and control regional and global plant invasions. No country in Central Asia has a national checklist of alien plants. A recent inventory counted 183 alien plant species in Kyrgyzstan, including archaeophytes and neophytes, established and casual. This preliminary checklist, which was developed for the Global Register of Introduced and Invasive Species in 2018, served as a starting point for the present study.

**New information:**

A complete inventory of *Xanthium* in Kyrgyzstan has revealed that three alien species are resident in the country. Their correct nomenclature is *X.orientale* (syn. *X.albinum*, *X.californicum*, *X.sibiricum* auct.; invasive neophyle of the period of extensive grain import to the USSR after the Second World War), *X.spinosum* (invasive neophyte of the period of the Second World War, which arrived as a contaminant on the relocated livestock) and *X.strumarium* (syn. *X.chinense*, *X.sibiricum*; archaeophyte of the Neolithic period, introduced with wheat cultivation, which had lost its invasive status and appeared on the verge of extinction when its pool was no longer renewed by contaminated grain). A history of introduction to Central Asia is uncovered for all the species of *Xanthium*. A further spread is documented for *Buniasorientalis*, with a new record extending its distribution to the Eastern Tian-Shan; a complex history of its introduction to Europe and Central Asia is inferred from the archaeological data and its recent dispersal, and the pathways of its introduction to Kyrgyzstan are established. *Erigeronannuus* s.str. is reported as new to Kyrgyzstan and Uzbekistan, and *E.lilacinus* as new to Kyrgyzstan, Uzbekistan, Kazakhstan, Nepal and Tajikistan (it was previously recorded as *E.annuus* s.l. from the three latter countries, in which the presence of *E.annuus* s.str. is not confirmed). These closely related species differ in their pathways of introduction and invasion status: *E.annuus* s.str. is an invasive established alien which was imported as a contaminant of forage, whereas *E.lilacinus* is mostly a casual (locally persisting) alien introduced with contaminated seed of ornamental plants or nursery material, and also intentionally introduced and locally established in the Botanical Garden in Bishkek. *Bidenstinctoria* (syn. *Coreopsistinctoria*) is newly recorded as a casual alien from a single locality in Kyrgyzstan; this species name is validly published here in conformity with the phylogeny of Coreopsideae.

Point maps of species distributions in Kyrgyzstan are provided on the basis of a complete inventory of the literature data, herbarium specimens and documented observations, and our recent fieldwork. The maps are documented with a dataset of herbarium specimens and observations. Period and pathways of introduction, vectors of dispersal, current and historical invasion status, evidence of impact and distributional trend are established or inferred for each species. Each species is discussed in the context of plant invasions in Central Asia as a whole.

These species accounts are part of the national database of alien plants which aims at producing a comprehensive overview and analysis of plant invasions in Kyrgyzstan.

## Introduction

Complete inventories of alien flora is a rather recent phenomenon. Even in relatively well-studied Europe, already by 2005, only very few countries had specialised checklists of alien plants, which would allow detection, analysis and control of plant invasions (Pyšek et al. 2009). The situation has been improved dramatically in the latest years, when a number of comprehensive and well-developed national checklists have been published (e.g. Arianoutsou et al. 2010, Verloove et al. 2020).

In Central Asia, alien plants have been considered part of floristic accounts and checklists and, therefore, have not been the subject of a separate study at the national scale. Nevertheless, although the floristic accounts usually highlighted alien plants or placed them separate from the native taxa, the non-native flora was largely neglected because of the common policy to omit mentions of rare or casual introductions. For this reason, complete and reliable data cannot be derived from the current synoptic botanical books, and special publications are required to compensate for this omission. So far, no such publication exists for any country of Central Asia.

Recently, during our work for the Global Register of Introduced and Invasive Species (Pagad et al. 2018), we have realised the need for new data collection towards national checklists and, ultimately, the database of alien plants of Central Asia. This work is viewed as an important complement to the existing floristic information, which is traditionally biased towards the native flora. Enhanced national checklists of alien plants are important instruments for scientists, conservationists and decision-makers, and making this information available online adds to the sustainability, ease of maintenance and global re-use of the work (Reyserhove et al. 2020).

Kyrgyzstan is a landlocked developing country of Central Asia, with a predominantly arid and highly continental climate and a mountainous terrain with a low (slightly over 5%) proportion of lowlands. Its territory totals 199,951 km^2^ (Otorbaev et al. 1962, Ryazantseva 1965, Ömürzakov 1987). The population density is rather low but growing rapidly (32 people per km^2^ in 2018). The dominating nation is the Kyrgyz, who were traditionally nomadic and, therefore, did not develop an old agricultural tradition; besides, agriculturally suitable lands occupy less than 7% of the territory. More extensive fields and orchard gardens were originally situated in the warm lowland parts of the country, the Chü Depression in the north and the Fergana Depression in the west, whereas crop cultivation and sown meadows became more widespread in the mountainous areas with the development of industrialisation in the Soviet times. Cultivation of ornamental plants is more recent and became widespread mostly during the 1950-1960s. Mining has always been important but became an industry in the beginning of the 20th century and has been an especially great part of the economy in the last 50 years, accounting for land disturbance and intensive transportation. This combination of a harsh climate, a complicated terrain, a low population density, a limited development of agriculture and a short history of industrial activities may account for the moderate amount of alien plants registered in the country, as can be seen below.

The landscape of Kyrgyzstan is composed of two main mountain systems (Tian-Shan and Hissar-Alay) and one main lowland depression (Fergana). These major landscape features define the plant diversity and distribution in the country. The Tian-Shan Mountain System can be subdivided botanically into three main parts (Sennikov and Tojibaev 2021): Western Tian-Shan, Northern Tian-Shan and Eastern Tian-Shan, which are well established and strictly defined regions in physical geography and plant geography. The Western Tian-Shan harbours the greatest plant diversity due to its unique combination of semidesert foothills and rather humid mountain ranges; besides the territory of Kyrgyzstan, it also includes parts of Kazakhstan, Tajikistan and Uzbekistan. The Northern Tian-Shan is also more humid but lacking the prominent arid component; it continues into China and Kazakhstan as the Dzungarian floristic subprovince of Grubov (1959). The Eastern Tian-Shan is prominently alpine and floristically poor; its Chinese continuation was called Kashgarian floristic subprovince by Grubov (1959). The Hissar-Alay Mountain System is bordering with the Pamir Mountain System along the state border of Kyrgyzstan with Tajikistan, which was marked along the prominently high watershed of the Transalay Range. Its northern part, delimited along the watershed of the Zeravshan Range, makes a vast share of its flora with the Tian-Shan and can be included into the latter following some schemes of physical geography (Merzlyakova 2002). This territory within Kyrgyzstan is designated here as Alay-Turkestan because of the inclusion of two main mountain ranges, the Alay and the Turkestan. To the same territory we add the southernmost, high-mountainous portion of the Fergana Range, which can be viewed as a longitudinal extension of the Alay; this part is devoid of steppe vegetation (Chupakhin 1964) and shares some peculiar plant species with the Alay (cf. Sennikov 2013). The arid lowlands of the Fergana Depression also impact the flora of Kyrgyzstan but cannot be geographically separated within the country because of a negligible proportion of its marginal parts being under the jurisdiction of Kyrgyzstan.

The flora of Kyrgyzstan includes nearly 4000 species of vascular plants, of which nearly 400 species (10%) are considered endemic to the country (Lazkov and Sultanova 2014).

The first data on the alien plants of Kyrgyzstan can be derived from the *Flora of the Kirghizian SSR* (Schischkin 1950, Vvedensky 1951, Schischkin 1952, Vvedensky 1953, Vvedensky 1955a, Vvedensky 1955b, Vvedensky 1957, Vvedensky 1959, Vvedensky 1960, Vvedensky 1962, Vvedensky 1965, Vykhodtsev 1967, Lebedeva 1970). However, this work made a poor distinction between native and alien plants, and the number of alien species recorded was very low.

A massive contribution to the weed flora of Kyrgyzstan was the work of Maria I. Deza. Many records and descriptions of alien vascular plants can be found in her books (Deza 1983, Deza 1989) and short reports (Deza et al. 1987, Deza 1988).

During the past 25 years, the alien plants of Kyrgyzstan have been studied by Georgy A. Lazkov. He published a long series of journal reports which contained numerous new records of alien plants (Lazkov 1997, Lazkov 1999, Lazkov 2001, Lazkov 2003, Lazkov 2007, Lazkov and Redina 2007, Lazkov et al. 2007, Lazkov and Ganybaeva 2008, Sennikov et al. 2011, Lazkov et al. 2011, Lazkov et al. 2012, German et al. 2013, Sennikov and Lazkov 2014, Lazkov et al. 2014b, Lazkov et al. 2014a, Lazkov and Sennikov 2015, Lazkov and Sennikov 2017). These reports provided a rich background material, but their contents were largely limited to the list of new records.

Most of these records were incorporated in the checklist of vascular plants of Kyrgyzstan (Lazkov and Sultanova 2011, Lazkov and Sultanova 2014). This checklist listed all the plants recorded from the country as spontaneous, including casual aliens. Alien plants (81 species) were noted as such, but this distinction was limited to neophytes, whereas archaeophytes (in this case, archaeophytes are plants that arrived because of human activities but became established before the beginning of the botanical exploration of the territory, i.e. before the 1860s) were considered native.

The first attempt to compile a comprehensive list of alien plants of Kyrgyzstan, which includes both neophytes and archaeophytes, was made by A. Sennikov and G. Lazkov in 2018 (Sennikov et al. 2021). In its current, revised form, the list contains 183 species (4.57% of the total flora) and provides a solid basis for the subsequent work on alien plants of the country.

The aim of our present work was to make a comprehensive inventory of records of non-native vascular plants in Kyrgyzstan (with publication of new records and previously inaccessible information), to place these records in the context of plant invasions in the world in general and in Central Asia in particular, and to establish or infer their invasion status and trends, with periods and pathways of their introduction. The purpose of this work was to collect the background information for the forthcoming analysis of plant invasions in Kyrgyzstan, which would help making informed administrative decisions to control plant invasions in order to reduce the risks posed to native ecosystems, agriculture and human well-being by invasive plants.

The information was processed and presented in the context of plant invasions in Central Asia as a uniformly structured, semantically rich taxonomic synopsis. This synopsis aims at providing the primary data for the future analysis of alien plants in Kyrgyzstan.

For the present contribution, we selected a number of taxa which were taxonomically confused or are new to the country or its parts. The complex of *Erigeronannuus* L. s.l. (Asteraceae) has been recently revised by Sennikov and Kurtto (2019), who provided an overview of its taxonomy, nomenclature, diagnostic characters, distribution, periods and pathways of introduction in Eastern Fennoscandia. This group has been largely neglected in Central Asia; its comprehensive review is presented here. The distinction between the native and secondary distribution areas of *Buniasorientalis* L. (Brassicaceae) used to be controversial (Laiviņš et al. 2006) but was recently resolved by genetic studies (Koch et al. 2017); we provide evidence for its current invasion to Central Asia. The taxonomy of *Xanthium* L. (Asteraceae) remained a nightmare until Tomasello (2018) provided a solid framework for its classification; this taxonomy and the recent advances in palaeobotany of the Last Postglacial in Asia allowed us to understand the times and causes of invasions of *Xanthium* in Central Asia. New casuals have been registered regularly in Kyrgyzstan and are also reported here (*Bidenstinctoria* (Nutt.) Baill. ex Sennikov).

## Materials and methods

The material is alphabetically organised (according to genera and species) as a taxonomic synopsis, with basic nomenclature (accepted names, basionyms and main synonyms, with references to the protologues), basic information on ecology (habitats in the native and secondary distribution areas) and biology (life form), extent of the native and secondary distribution areas, with emphasis on Central Asia and Kyrgyzstan, details pertinent to the introduction in Central Asia in general (politically defined as the whole territory of Kazakhstan, Kyrgyzstan, Tajikistan, Turkmenistan and Uzbekistan) and Kyrgyzstan in particular (period and pathways of introduction, vectors of dispersal, current and historical invasion status, evidence of impact for agriculture, native ecosystems and urban areas, and trends), and also taxonomic and nomenclatural comments when deemed relevant.

We determined the status of alien plant species following the definitions proposed by Richardson et al. (2000). We used the actual and historical data (herbarium specimens, palaeobotanical records, published historical information: Webb 1985) in order to uncover the history and pathways of introduction of alien plants species to the study area. The pathways of introduction were coded according to Hulme et al. (2008) and interpreted as recommended by Harrower et al. (2018). We used major events of the recent political history of Kyrgyzstan (1876, Russian conquest; 1917, revolutions in Russia; 1941-1945, USSR in the Second World War; 1991, independence from the USSR) to define the periods of introduction. Trends were inferred on the basis of observations made during the last 20 years.

**Figure 1.**
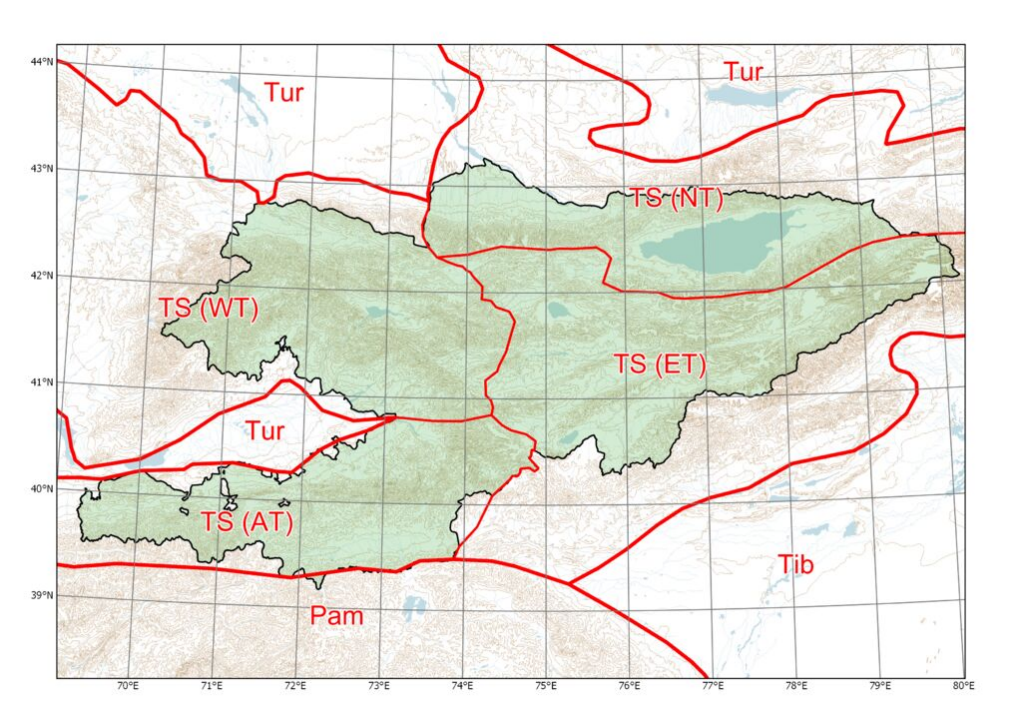
Major phytogeographic regions of Kyrgyzstan. Divisions (thick lines): TS (Tian-Shan), Tib (Tibet), Tur (Turanian), Pam (Pamir). Subdivisions (thin lines): AT (Alay-Turkestan), ET (Eastern Tian-Shan), NT (Northern Tian-Shan), WT (Western Tian-Shan).

Species distributions in Kyrgyzstan were formalised on the basis of phytogeographic regions. Instead of the system of phytogeographic divisions developed by Rudolf V. Kamelin (Kamelin 1973, Kamelin 2002), which was based on the main features of vegetation, we used a phytogeographic subdivision of mountainous areas as described in the Introduction, with three main regions in the Tian-Shan area and one main region in the Hissar-Alay area (Fig. 1). This scheme is based on the features of physical geography and plant diversity, taking into account the patterns of narrow endemism and closely related taxonomic groups.

Species records in Kyrgyzstan were traced as completely as possible on the basis of herbarium specimens and documented observations, with the addition of the authors' field observations. The collections of main Herbaria, in which specimens from the country are known to have been deposited (FRU, H, LE, MW, TASH), were screened, and the taxonomic identity of each specimen was re-evaluated. The specimens were examined mostly *de visu*, but partly from digital images. Further records were traced from observations published through citizen science platforms (iNaturalist 2021, Plantarium 2021). When original georeferences were not available, records were georeferenced *ad hoc* using contemporary and modern topographic maps. Transcripts of the geographic data were standardised according to Ömürzakov et al. (1988) and translated into English.

The cumulative dataset of all the records collected for the present work was published separately through GBIF (Sennikov and Lazkov 2021). The occurrences used in the present contribution are also available as Suppl. material 1. Distributional maps were created on the basis of these records.

Species distributions in Central Asia were traced from published sources and major herbarium collections (LE, MW, TASH). Reliable published records obtained from literature, specimens deposited at LE and TASH and those available through the digital collections of ALTB (Vaganov et al. 2021), MW (Seregin 2021) and UFU (Tretyakova et al. 2021), as well as observations available through citizen science platforms (iNaturalist 2021, Plantarium 2021) were taken into account.

The information about species distributions outside Central Asia was derived from PoWo (2021) and various taxonomic and floristic accounts.

The taxonomy and nomenclature are original and were largely published in Sennikov and Tojibaev (2021). Further taxonomic and nomenclatural decisions were made in separate accounts (Sennikov and Kurtto 2019) and also here.

## Taxon treatments

### Bidens tinctoria

(Nutt.) Baill. ex Sennikov 2021

7FB1D4D3-6B62-5920-A322-DFFB10B8DD7C

urn:lsid:ipni.org:names:185283-1

Bidens tinctoria (Nutt.) Baill. [Hist. Pl. (Baillon) 8: 305 (1882)] ex Sennikov, **comb. nova** — *Coreopsistinctoria* Nutt., J. Acad. Nat. Sci. Philadelphia 2: 114 (1821).

#### Diagnosis

The species can be easily recognised by its ray flowers, which are typically yellow with a large red spot at the base, but sometimes purely yellow or red. Leaves are nearly sessile, pinnately divided with long and narrow lateral lobes.

#### Distribution

##### Native distribution

This species is native to North America (from southern Canada to northern Mexico) (Strother 2006).

##### Secondary distribution

Neophyte in Central America, Europe, Western and Southern Asia, Southern Africa.

##### Distribution in Central Asia

First recorded as an alien in Kyrgyzstan here.

This species was common in ornamental cultivation in Uzbekistan already by the 1960s, although not reported as escaped from cultivation (Nabiev 1962a). However, the contemporary literature on the flora of Kyrgyzstan (Sultanova 1963, Sultanova 1965) made no mention of the species, probably because of confusion with *Cosmossulphureus* Cav. (cf. Verloove and Lambinon 2008). Neither was it mentioned in the latest manual of the Central Asian flora (Adylov and Zuckerwanik 1993), apparently due to the lack of spontaneous records.

**Figure 2.**
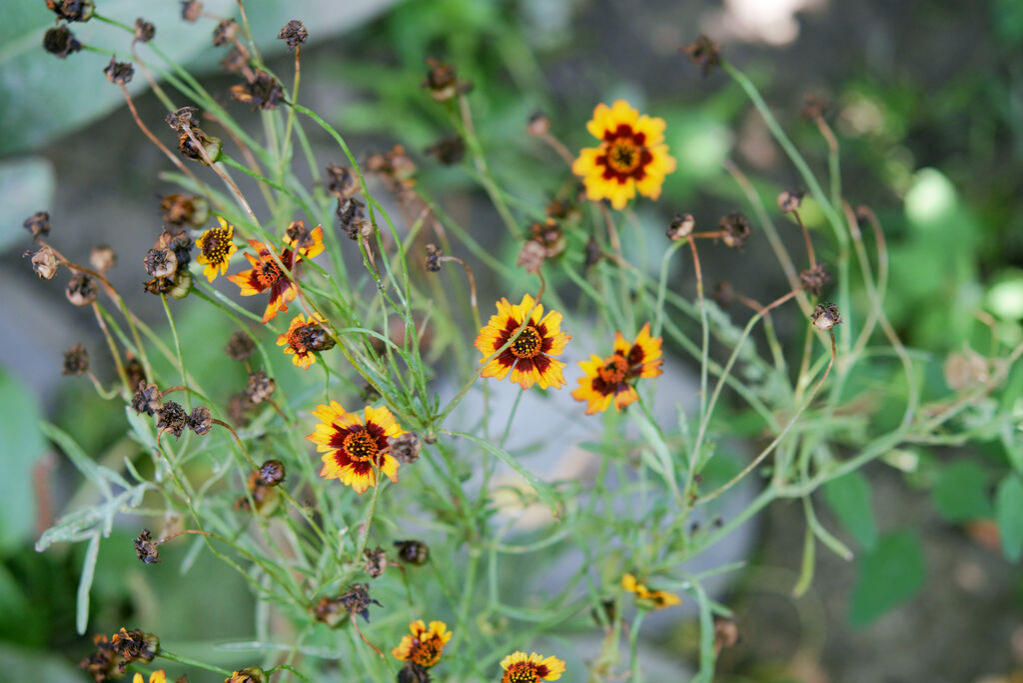
*Coreopsistinctoria* in cultivation in Kyrgyzstan (photo by G. Lazkov, September 2021).

Currently the species was observed in ornamental cultivation in Kyrgyzstan (Fig. 2).

##### Distribution in Kyrgyzstan

**Figure 3.**
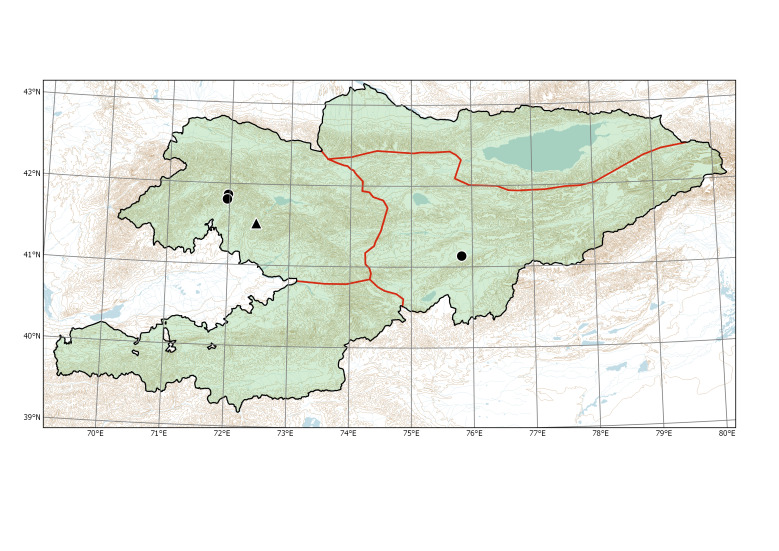
Distribution of *Bidenstinctoria* (triangle) and *Buniasorientalis* (dots) in Kyrgyzstan.

Western Tian-Shan (Fig. 3).

We discovered this species once and for the first time in 2016. A few flowering individuals were observed in ruderal places around an isolated gasoline station on the main road along the Naryn River in Jalal-Abad Region, at an elevation about 800 m a.s.l.

#### Ecology

Prairies, on moist, sandy or clayey soils in the native distribution area (Strother 2006); disturbed places and waste ground in the secondary distribution area.

#### Biology

Annual.

#### Notes

The taxonomy of Coreopsideae Lindl. has been controversial since the original description of its main genera, *Bidens* L. and *Coreopsis* L. Tadesse et al. (1996) stressed that the main diagnostic characters traditionally used to delimit these genera (achene awns and wings) are unreliable because of the presence of intermediate states and geographic disparity; the differences in plant habit were used to support the other characters. Many studies (e.g. Mort et al. 2008, Knope et al. 2020) demonstrated that both *Bidens* and *Coreopsis* are polyphyletic and resolved as a number of clades intermixed with each other. Since many African (but not American) species of *Coreopsis* have already been reclassified in *Bidens*, Banfi et al. (2017) completed such transfers for the Euro+Med area. We agree that *Bidens* and *Coreopsis* are not recognisable by morphology and cannot be maintained on phylogenetic grounds and, therefore, accept *Bidens* as a single broadly defined genus.

The transfer of *Coreopsistinctoria* Nutt. was commonly attributed to Baillon (1882). In this book, the taxonomic placement of *Coreopsis* as a section of *Bidens* was suggested and some constituent species were listed including *Coreopsistinctoria*. Since the combination "*Bidenstinctoria*" was only implied but did not appear in print in that text, it was not validly published according to Art. 35.2 of the ICN (Turland et al. 2018). It was not inadvertently validated later by Jackson (1893), who indexed this species name but typeset it in Italics and, therefore, indicated its taxonomic status as a synonym (Greuter 1985). It was not validly published by Banfi et al. (2017) because these authors did not provide a full and direct reference to the basionym publication. Since we cannot trace any other acceptance of this binomial in botanical literature, we assume that it remains invalidly published. For this reason, this species name is treated as a new combination here.

In the past, *Coreopsisbasalis* (A.Dietr.) S.F.Blake (syn. *C.drummondii* (D.Don) Torr. & A.Gray) was reported as the only species of this genus present in ornamental cultivation in Kyrgyzstan (Sultanova 1965). This species is immediately distinct from *C.tinctoria* in its much wider, elliptic to lanceolate leaf lobes (Strother 2006). So far, *C.basalis* has never been reported as escaped from cultivation in Kyrgyzstan.

#### Introduction to Kyrgyzstan

##### Period of introduction

Neophyte.

We collected the species for the first time in 2016. This introduction falls within the period of independence of Kyrgyzstan (since 1991).

##### Pathways of introduction

Transport - Contaminant: Food contaminant (including of live food).

This species is a popular ornamental plant, which was widely cultivated in Central Asia (data from Uzbekistan) already by the 1960s (Nabiev 1962a). However, no evidence of any ornamental cultivation was observed at the time of our record.

The ruderal ground, on which the species was seen in Kyrgyzstan, has been used as a parking and turning place for long-distance trucks and other transport. Since the species is known as a crop weed in North America (Everitt et al. 2007) and has been recorded as having arrived with contaminated grain in Europe (e.g. Suominen 1979, Borisova and Golubeva 2006, Verloove et al. 2020, Verloove 2021), we assume the same pathway of introduction also occurred in our locality.

Further dispersal does not take place.

##### Invasion status

Casual (ephemeral, no viable population observed).

##### Evidence of impact

Agriculture - no impact (not observed as a weed). Native ecosystems - no impact (not observed in native habitats). Urban areas - minor impact (ruderal occurrence, casual).

##### Trend

No expansion observed, no dynamics known.

### Bunias orientalis

L. 1753

5AC35411-F8A8-5070-947E-CA2522C5743D

urn:lsid:ipni.org:names:279703-1

Bunias orientalis L., Sp. Pl. 2: 670 (1753).

#### Distribution

##### Native distribution

Eastern Europe (southern parts up to the boreal zone), Asia (Western Caucasus, Transcaucasia, eastern Anatolia). Two main parts of the distribution area, Eastern Europe and the Caucasus, correspond to two main gene pools (Koch et al. 2017). The hypothesis of its non-native origin in Europe (Meusel and Jäger 1965) should, therefore, be rejected.

##### Secondary distribution

Neophyte and archaeophyte in Europe (outside its south-eastern part) and Northern Asia, neophyte in Central Asia, China and North America.

Since the 19th century, the species has been dispersed throughout other parts of Europe and, since the 20th century, also in Asia. Its early introduction to France was frequently ascribed to military activities of the Russian army during the War of the Sixth Coalition (1813-1814); this legendary report first appeared in an early German textbook (Endlicher and Unger 1843), was subsequently promoted in popular literature (Rachinsky 1866) and finally entered academic writing (Klinge 1887). According to the original source (Loiseleur Deslongchamps 1807), the plant was actually naturalised from "garden" (i.e. experimental) cultivation in three places near Paris well before the War. Its earliest introductions to Europe seem to have been regularly linked with its cultivation for fodder or salad (Curtis 1812, Sinclair 1825, Lawson and Lawson 1836), which was followed by a massive invasion with imported crop seeds and fodder (e.g. Suominen 1979, Pyšek et al. 2017). The species became a noxious weed and invasive in Northern Europe (Scandinavia and Finland) already in the second part of the 19th century (e.g. Fries 1845, Woll 1899). Its recent spread in Europe is linked with transportation of contaminated grain and fodder in the second part of the 20th century (Jehlík and Slavík 1968, Jehlík and Hejný 1974, Suominen 1979), and its local dispersal may occur by vehicles (Kiełtyk 2014).

Besides the history of introduction in the modern period (neophyte records), archaeological evidence indicates that *Buniasorientalis* was cultivated in Europe (Poland) as early as in the 12th and 13th centuries, most likely for food and fodder, and may remain locally surviving since then (Celka 2011).

##### Distribution in Central Asia

Kazakhstan, Kyrgyzstan, Uzbekistan.

This species was originally introduced to Central Asia (eastern Kazakhstan) and southern Siberia as food by nomadic Turkic people over 2300-2400 years ago (Dashkovsky et al. 2014), but this introduction had been eventually extirpated as no early botanical records indicated the presence of this species more easterly from the south-eastern Urals (Ledebour 1841). The first recent record of the species more easterly of the Urals, in southern Siberia, is dated 1912 (Krylov 1931); the plants were collected as crop weeds and along roadsides, and the species was apparently introduced as a crop seed contaminant when the agrarian colonisation of Siberia was intensified by the Department of Migrations (1896-1917). This introduction occurred from East European populations of the species (Koch et al. 2017).

*Buniasorientalis* was first known from Kazakhstan (as ruderal in the eastern and south-eastern parts and in the Transili Alatau) (Vasilieva 1961, Vasilieva 1969, Nabiev 1974). This distribution pattern (several records in the easternmost hilly part of the country and single records in the mountains) is still valid (Plantarium 2021). According to herbarium collections kept at LE, the first specimen of the species was collected from Kazakhstan in 1960, but its first records are apparently earlier.

The species was introduced to Uzbekistan (Tashkent Region, Boʻstonliq District) from Eastern Europe and was found locally established already in 1973 (Koch et al. 2017), but this record remained formally unpublished and was not taken into account in any other literature.

We discovered this species in Kyrgyzstan in 2009, for the first time in the Sary-Chelek Nature Reserve (Lazkov et al. 2011). One more locality was found in 2021.

##### Distribution in Kyrgyzstan

Western Tian-Shan, Eastern Tian-Shan (new record) (Fig. 3).

**Figure 4.**
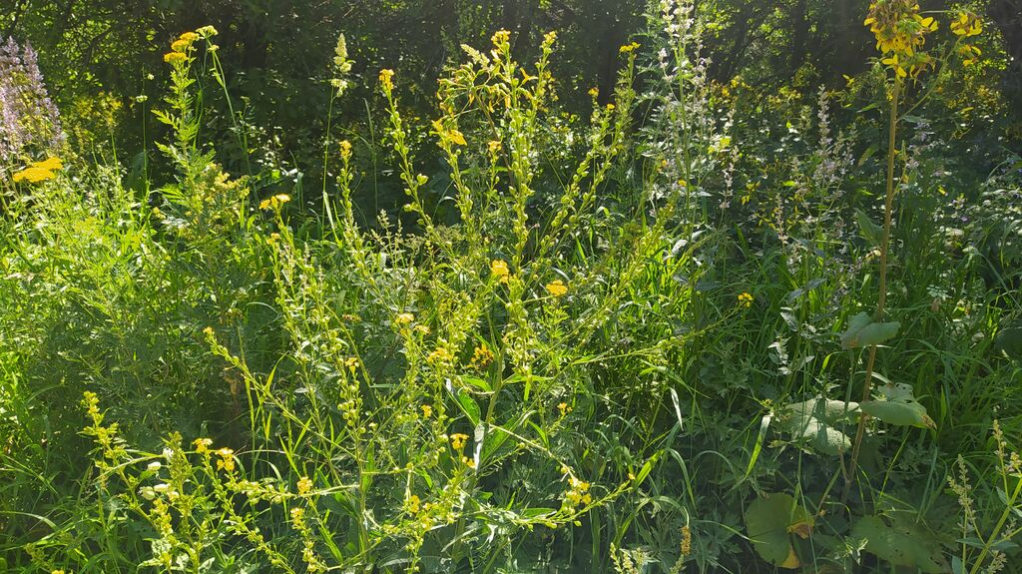
Plants of *Buniasorientalis* in the Sary-Chelek Nature Reserve (photo by G. Lazkov, 8 July 2021).

So far, the species is known from two remote territories. In the Sary-Chelek Nature Reserve, it was first discovered (Lazkov et al. 2011) as a large population along the side of the road leading from Arkyt Village to Lake Sary-Chelek (Fig. 4). Since 2018, the species was registered also in Arkyt Village, to which it was transported with hay from managed meadows (Lazkov, pers. obs.).

In 2021, a large population of *Buniasorientalis* was found at Acha-Kayyingdy Village (At-Bashy Mountain Range), on a fallow field with ruderal vegetation. Its further occurrence in the country can be predicted on cultivated lands.

In Kyrgyzstan, the species occurs at elevations between 1800 and 2200 m, which are suitable for crop and forage production and correspond to altitudes in the native distribution area of the species.

#### Ecology

Mountain meadows at altitudes up to 2500 m in the native area; managed and natural meadows, fallow lands, pastures, ruderal places and roadsides with preference for disturbed ground in the secondary area.

The species has been a common weed of spring crops in Eastern Europe (Jarmolenko and Vasilchenko 1934) and was considered a common contaminant of crop seed and a noxious weed in Finland (Woll 1899) and Sweden (Fries 1845) already by the mid-19th century, due to the import of Russian rye.

#### Biology

Perennial forb with biennial stems and a strong taproot. Promoted by disturbance and moving, with very high generative effort (Steinlein et al. 1996, Woitke and Dietz 2002).

#### Introduction to Kyrgyzstan

##### Period of introduction

Neophyte.

The first record is dated 2009 (Lazkov et al. 2011). We feel confident that this conspicuous species was not overlooked in the times of the Soviet botanical exploration (especially considering that its first record came from the most actively explored area) and had arrived during the period of the independence of Kyrgyzstan (since 1991).

##### Pathways of introduction

Transport - Contaminant: Seed contaminant. Transport - Contaminant: Contaminated bait.

According to the publicly available information (calls for tenders), the Sary-Chelek Nature Reserve regularly (nowadays twice a year) purchases considerable amounts of fodder to feed wild animals. This fodder has been imported from Russia, where *Buniasorientalis* is a common weed and distributed for animal consumption across the territory of the Nature Reserve. Further dispersal occurred by hay management.

In the second locality at Acha-Kayyingdy, the species was apparently a crop weed, thus being a contaminant of crop seed.

##### Invasion status

Locally naturalised, potentially invasive.

##### Evidence of impact

Agriculture - moderate impact (weed of fallow fields and managed meadows; limited occurrence). Native ecosystems - minor impact (on managed meadows). Urban areas - minor impact (occurrence in ruderal places and on roadsides).

##### Trend

Increasing (observed).

### Erigeron annuus

(L.) Desf. 1804

42AE86FA-FE38-56FC-9973-73C1BFA2A3C9

urn:lsid:ipni.org:names:77216525-1

Erigeron annuus (L.) Desf., Tabl. École Bot.: 102 (1804) — *Asterannuus* L., Sp. Pl. 2: 875 (1753) — *Phalacrolomaannuum* (L.) Dumort., Fl. Belg.: 67 (1827) — *Stenactisannua* (L.) Nees, Gen. Sp. Aster.: 273 (1832).
Erigeron annuus Erigeronramosusvar.septentrionalisErigeronannuussubsp.septentrionalisStenactisannuasubsp.septentrionalis*Stenactisseptentrionalis*Phalacrolomaannuumsubsp.septentrionale*Phalacrolomaseptentrionale*

#### Diagnosis

In the group of *Erigeronannuus* s.l., *E.annuus* s.str. can be distinguished by its narrower cauline leaves with less prominent teeth, white ray flowers and involucres with hairs 0.8–1.2(1.5) mm long (Sennikov and Kurtto 2019).

#### Distribution

##### Native distribution

North America (Canada, USA, Mexico).

##### Secondary distribution

Neophyte in Europe and Asia. In Europe, this species belongs to the most widely distributed alien vascular plants (Lambdon et al. 2008). It is also listed among the most dangerous invasive species in Russia (Dgebuadze et al. 2018).

##### Distribution in Central Asia

Kyrgyzstan, Uzbekistan.

Tulaganova (1993) reported *Erigeronannuus* s.l. from Almaty City, Kazakhstan. This was the first record of this species complex published in Central Asia. A recent record from the same area, situated close to the Botanical Garden and the National University (iNaturalist 2021), shows that this population belongs to *E.lilacinus* as defined by Sennikov and Kurtto (2019).

In Tajikistan, this species, broadly defined following Frey et al. (2003), was reported from Dushanbe City (Nobis et al. 2017). The plants photographed in Dushanbe by Dzhamshed Sattarov (Plantarium 2021) have broad and coarsely dentate leaves and lilac flowers and, therefore, correspond to *E.lilacinus* (Sennikov and Kurtto 2019).

Our new reports of this species (as currently defined) provide its first reliable records from Central Asia and extend its known secondary distribution to Kyrgyzstan and Uzbekistan.

In Uzbekistan, the species was first recorded by Tulkin Tillaev in 2020 from meadows in Ulug'bek District of Tashkent City (Plantarium 2021). It forms a large population, which is apparently established.

##### Distribution in Kyrgyzstan

**Figure 5.**
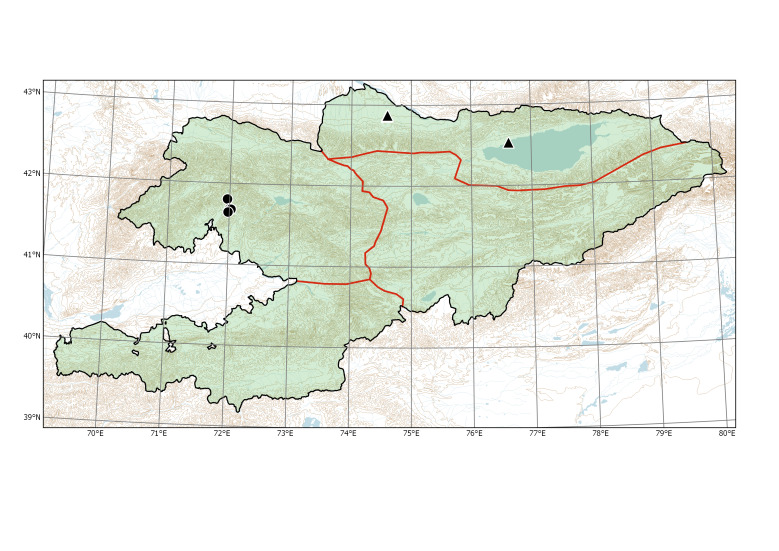
Distribution of *Erigeronannuus* (dots) and *E.lilacinus* (triangles) in Kyrgyzstan.

Western Tian-Shan (Fig. 5).

**Figure 6.**
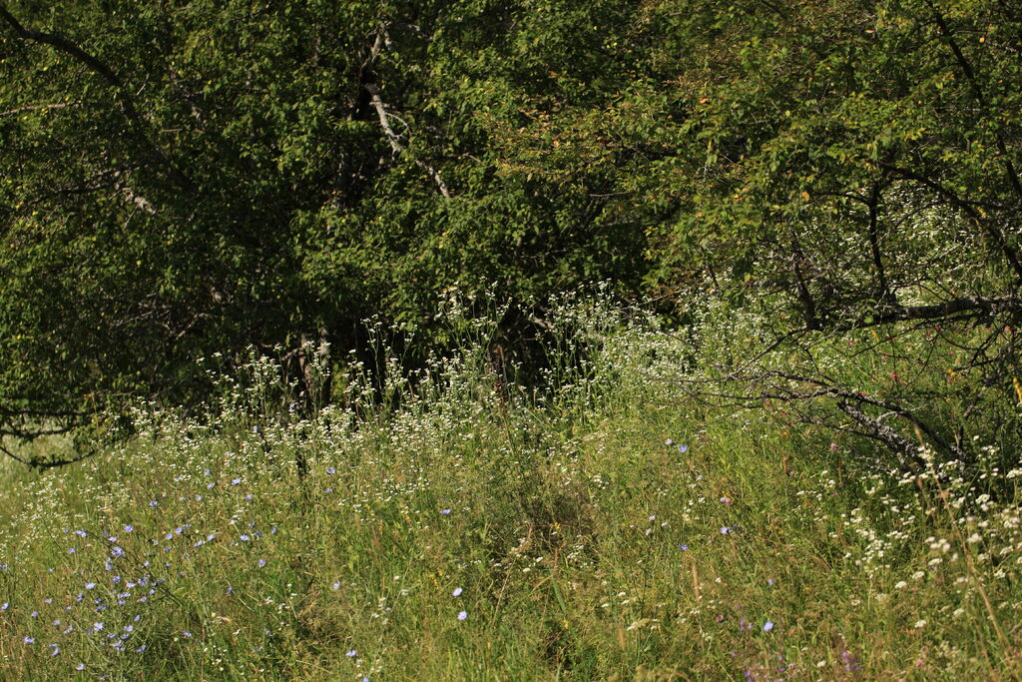
Large stands of *Erigeronannuus* s. str. in the Sary-Chelek Nature Reserve (photo by A. Sennikov, 12 July 2016).

We found large populations of this species along the Avletim River (downstream from Avletim Village) and the Kojo-Ata River (downstream from Arkyt Village up to the Avletim River), including the vicinities of Arkyt Village. The populations around Arkyt (Fig. 6) have been observed during multiple visits since 2009. In this area, the species is connected with managed meadows (used for hay-making) but occurs extensively also on natural meadows along river sides.

As observed in the Caucasus, the invasion of *Erigeronannuus* may be highly aggressive on hay meadows and pastures of mountainous areas up to high elevations (Pshegusov et al. 2020), and its further spread in Kyrgyzstan is therefore expected.

In the Sary-Chelek Nature Reserve, the species was found at elevations between 1000 and 1300 m, which are optimal for grasslands and forb meadows. The upper altitudinal limit observed at 1000 a.s.l. in the Swiss Alps in Europe (Trtikova et al. 2010) is not valid in the Tian-Shan Mountains.

#### Ecology

Prairies and meadows in the native distribution area; managed meadows and ruderal places in the secondary distribution area. In Europe, this species belongs to the three most invasive neophytes occurring in natural and semi-natural grasslands (Axmanová et al. 2021).

#### Biology

Annual or biennial. The species is characterised by very high seed productivity (Stratton 1991) and easily colonises bare or disturbed grounds (Stratton 1992). The seeds of *Erigeronannuus* were found to inhibit the development of seedlings of some other species (Oh et al. 2002, Kudryavtseva et al. 2020).

#### Notes

The taxonomy, nomenclature, native and secondary distributions of species-level taxa in the complex of *Erigeronannuus* s.l. were resolved by Sennikov and Kurtto (2019).

#### Introduction to Kyrgyzstan

##### Period of introduction

Neophyte.

The species was first recorded in the wild in 2009 and had apparently arrived during the period of the independence of Kyrgyzstan, in the 2000s.

##### Pathways of introduction

Transport - Contaminant: Contaminated bait.

Similarly to *Buniasorientalis*, this species was seemingly introduced to the Sary-Chelek Nature Reserve as a contaminant of imported fodder from Russia, where it is known as one of the most widely distributed invasive plants (Vinogradova et al. 2018). This species is highly favoured by mowing, which promotes its local invasion (Song et al. 2018); that was apparently the case in Kyrgyzstan, where it was unintentionally dispersed by the inhabitants of Arkyt Village, who used the territory intensely for hay-making (Sennikov & Lazkov, pers. obs.).

Further dispersal occurs by wind and human management.

##### Invasion status

Fully naturalised, highly invasive.

##### Evidence of impact

Agriculture - moderate impact (weed of pastures and hay-making meadows, with limited distribution; not recorded in crop production areas). Native ecosystems - moderate impact (invading riversides, grasslands and other meadows, with limited distribution). Urban areas - minor impact (very rare ruderal plant in populated places, including private gardens).

##### Trend

Increasing (observed).

### Erigeron lilacinus

(Sennikov & Kurtto) Sennikov 2020

E79301DC-4BA8-5FB9-A017-966D103F9C36

urn:lsid:ipni.org:names:77216014-1

Erigeron lilacinus (Sennikov & Kurtto) Sennikov, Wulfenia 27: 2 (2020) — Erigeronannuussubsp.lilacinus Sennikov & Kurtto, Memoranda Soc. Fauna Fl. Fenn. 95: 47 (2019).

#### Diagnosis

In the group of *Erigeronannuus* s.l., *E.lilacinus* can be distinguished by its broader cauline leaves with more prominent teeth, pale lilac ray flowers, and involucres with hairs 0.8–1.2(1.5) mm long (Sennikov and Kurtto 2019).

#### Distribution

##### Native distribution

North America (south-eastern Canada, north-eastern and eastern USA).

##### Secondary distribution

Neophyte in Europe and Asia.

In Eastern (Tropical) Asia, the occurrences of this species were known from Taiwan and Vietnam (Sennikov et al. 2020). Both records previously reported from Nepal (Sukhorukov 2015) also belong to *E.lilacinus* because of the lilac ray florets and coarsely dentate leaves.

The presence of the species in Central Asia is reported for the first time here. The distribution in other Asian countries has not been studied yet.

##### Distribution in Central Asia

First reported from Kazakhstan, Kyrgyzstan, Tajikistan and Uzbekistan here.

In Uzbekistan, the species was first recorded by Alexander Sukhorukov in 2001 at the entrance to the Botanical Garden in Tashkent, where it occurred abundantly on ruderal places (Seregin 2021). Tulkin Tillaev (and Alim Gaziev) also recorded this species in 2012 from ruderal places in Ulug'bek District of Tashkent City (Plantarium 2021). The species is considered casual but locally persisting, on the way to naturalisation.

In Tajikistan, the species was previously reported as *E.annuus* [s.l.] (Nobis et al. 2017). The plants were collected in 2007 (Nobis et al. 2017) and observed in 2016 (Plantarium 2021) along the streets, probably introduced as weeds of ornamental cultivation.

In Kazakhstan, the species is known from ruderal places and as a weed of flower beds in Almaty City. Two recent records are known: from the area situated close to the Botanical Garden and the National University, by Ruslan Nurkhanov in 2020 (iNaturalist 2021), and from unspecified vicinities of the city in the Transili Alatau, by Igor Syazhkin in 2010 (Plantarium 2021). The oldest record from the same city (Tulaganova 1993), which was published as *E.annuus* [s.l.], has not been verified.

##### Distribution in Kyrgyzstan

Northern Tian-Shan.

**Figure 7.**
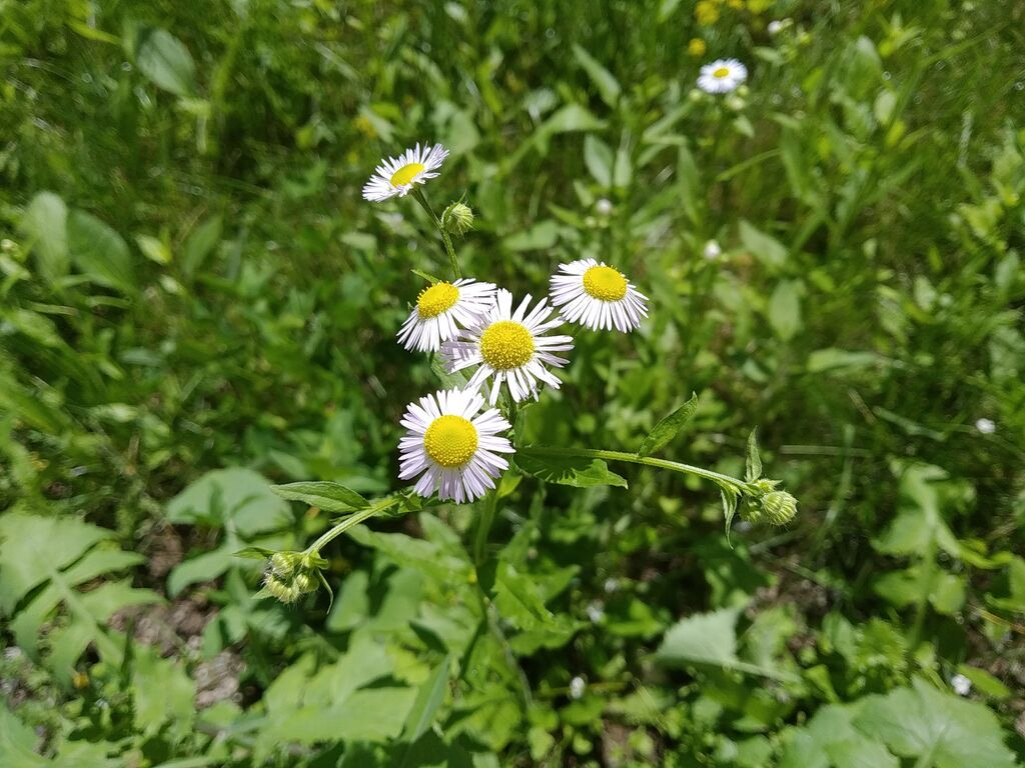
*Erigeronlilacinus* on flower beds in Bishkek (photo by G. Lazkov, 11 June 2020).

The species is known from two populated places. In Bishkek, it has been recorded since the 1980s as having escaped from cultivation and then as fully naturalised in the Botanical Garden of the National Academy of Sciences (I. Popova, pers. comm.). Besides, recently it was found in two more places in Bishkek, by Galina Chulanova in 2015 (ca. five flowering individuals) on street lawns situated near the Botanical Garden (Plantarium 2021) and by Georgy Lazkov in 2020 (ca. 10 individuals) on flower beds situated close to the Panfilov Park (Fig. 7), and also recorded from one place in a popular resort area along the northern side of Lake Ysyk-Köl, where it was observed by Galina Chulanova in 2011 (Plantarium 2021). The latter area is known for numerous introductions of ornamental plants.

#### Ecology

Prairies and meadows in the native distribution area; artificial meadows, ruderal places and cultivated lands in the secondary distribution area.

#### Biology

Annual or biennial.

#### Introduction to Kyrgyzstan

##### Period of introduction

Neophyte.

In the Botanical Garden, the species was intentionally introduced in the 1970s and became established in the 1980s, during the late Soviet period. The species was unintentionally introduced in the 2000s (first recorded in 2011), during the period of the independence of Kyrgyzstan.

##### Pathways of introduction

Transport – Contaminant: Contaminant nursery material. Escape from confinement: Botanical garden.

In agreement with observations of Sennikov and Kurtto (2019), in Kyrgyzstan, *Erigeronlilacinus* was recently found in places of cultivation of ornamental plants or on artificial lawns. Consequently, we consider the species to have arrived with contaminated seed of ornamental plants or nursery material.

The record in the Botanical Garden in Bishkek has a different origin. In that place, the species was intentionally introduced for experimental cultivation in the 1970s (erroneously as "*Conyzacanadensis*") but quickly spread out of control and became established already during the 1980s. Currently, it is fully naturalised in the Garden but is still kept within its limits, except for a few cases of intentional introduction or unintentional secondary dispersal to private gardens (I. Popova, pers. comm.).

##### Invasion status

Casual, temporarily persisting or locally established, not invasive. So far, we have no evidence that the species formed stable populations rather than short-lived local colonies in the places of its accidental introduction, and no further dispersal from those places was observed. However, *Erigeronlilacinus* has recently become abundant in man-made habitats (especially fallow and abandoned fields) in Central Russia (Sennikov, pers. obs.) and may, therefore, become invasive also in Kyrgyzstan. Its population in the Botanical Garden is locally naturalised and may potentially serve as a source of invasion in the future, as evident from some occasional instances of secondary dispersal.

##### Evidence of impact

Agriculture - no impact (not recorded in crop production areas). Native ecosystems - no impact (restricted to populated places). Urban areas - minor impact (very rare weed of ornamental gardens and street lawns, also as a ruderal plant).

##### Trend

Increasing (observed).

### Xanthium orientale

L. 1763

B168632F-72A5-5042-80E7-F8755DD83780

urn:lsid:ipni.org:names:260872-1

Xanthium orientale L., Sp. Pl., ed. 2, 2: 1400 (1763).
Xanthium orientale *Xanthiumitalicum*Xanthiumstrumariumsubsp.italicumXanthiumorientalesubsp.italicum
Xanthium orientale *Xanthiumbrasilicum*
Xanthium orientale *Xanthiumcalifornicum*Xanthiumorientalesubsp.californicum
Xanthium orientale *Xanthiumalbinum*Xanthiumripariumvar.albinum

#### Diagnosis

This species is characterised by unarmed leaves and narrowly cylindrical hairy burrs 2-3 cm long with numerous, closely spaced hooked prickles.

#### Distribution

##### Native distribution

This species is native to North America and South America (Löve and Dansereau 1959).

##### Secondary distribution

Neophyte in Europe, Mediterranean, South Africa, Western, Boreal, Central and Tropical Asia, Australia.

In arid regions of Asia, the species was known from several localities in Iran already 40 years ago (Dittrich 1989). In China, it was recorded for the first time in Beijing in 1991 (as *X.italicum*: Xu et al. 2012). In Russian Asia, it is known from many localities in southern Siberia (Khanminchun 1998) and the southern Far East (Kozhevnikov and Kozhevnikova 2011).

##### Distribution in Central Asia

Kazakhstan, Kyrgyzstan, Tajikistan, Uzbekistan.

In Kazakhstan (as *X.albinum*: Ebel and Ebel 2003), it was first found as ruderal in Tekeli Town (Almaty Region; Dzungarian Alatau), Öskemen [Ust-Kamenogorsk] Town and north-west of Semei Town (East Kazakhstan Region; Altay Mts.); the first record (specimen dated 1955) was registered near Semei Town. Its latest records from the lowland parts of the country (Qarağandy Region, Aqtoğai District, riversides and ruderal places; Pavlodar Region, Ekіbastūz Town, ruderal places; Túrkistan, Qyzylorda and East Kazakhstan Regions) indicate a greater distribution (as *X.albinum*: Nobis et al. 2015, Ebel et al. 2016, Plantarium 2021).

In Tajikistan (as *X.californicum*: Kinzikaeva 1988, Nabiev 1993), the species was recorded as a ruderal from two places, at Zafarobod Town and Dushanbe City.

In Uzbekistan (as *X.californicum*: Nabiev 1993; as *X.albinum*: Esanov 2016), the species was known as a weed in Tashkent (first record dated 1986) and as widely naturalised on cotton fields, fallow fields and roadsides of the Buxaro Agricultural Oasis (first recorded in 2007 but apparently established long before). Further records (Plantarium 2021) suggest its broad distribution in the Fergana Depression. The high impact of the species on cotton fields in Uzbekistan (Esanov 2016) agrees with the reports from the USA (Weaver and Lechowicz 1983), but a diversity of Central Asian reports from ruderal habitats suggests its multiple introductions not limited to the cotton cultivation.

Formally reported for the first time from Kyrgyzstan here.

The first record of the species from Central Asia (dated 1955) seems to be linked with the infamous Virgin Lands campaign, which started in 1954 as the extensive cultivation of previously uncultivated lands in northern Kazakhstan. To increase the yield of wheat crops, quality seed of productive varieties of American origin seem to have been partly used.

The large-scale invasion of *X.orientale*, however, occurred later, in the 1960s, when the extensive import of North American (largely Canadian) grain (wheat and maize) started to compensate for the shortage of domestic grain and fodder (Chistyakov 2009, FAO 2021).

##### Distribution in Kyrgyzstan

**Figure 8.**
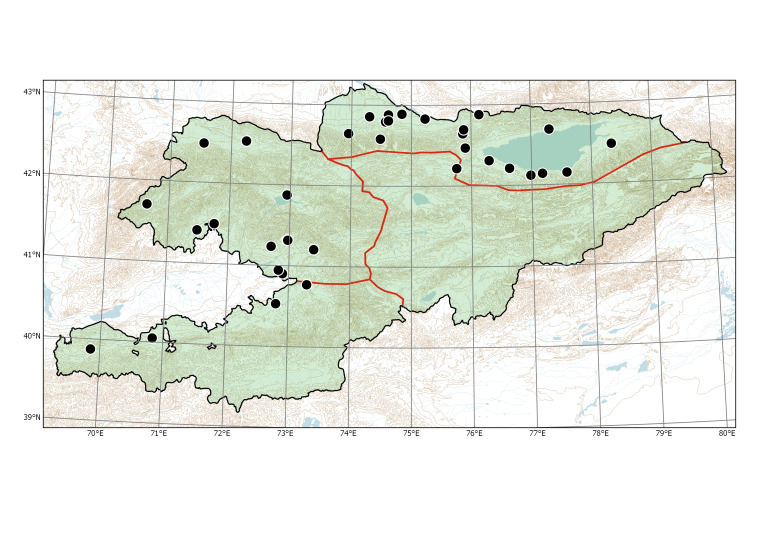
Distribution of *Xanthiumorientale* in Kyrgyzstan according to herbarium specimens and our observations.

Western Tian-Shan, Northern Tian-Shan, Alay-Turkestan (Fig. 8).

In Kyrgyzstan, this species was rather neglected. Deza (1983) clearly distinguished it from the typical *X.strumarium* L. and stated that it was found most commonly in (but not restricted to) the Chü Depression, where it occurred along roadsides and irrigation ditches, in populated places, on fallow fields and field margins, in gardens and on cultivated fields. Deza (1983) misapplied the name *X.sibiricum* Patr. ex Widder, which she seemingly borrowed from Smolianinova (1959) through Gorbunova (1965), to this species in spite of their contrasting morphology, thus assuming its native distribution area stretching from Siberia to Central Asia. Due to this incorrect nomenclature, the species was omitted from the recent checklist of the flora of Kyrgyzstan (Lazkov and Sultanova 2011, Lazkov and Sultanova 2014).

**Figure 9.**
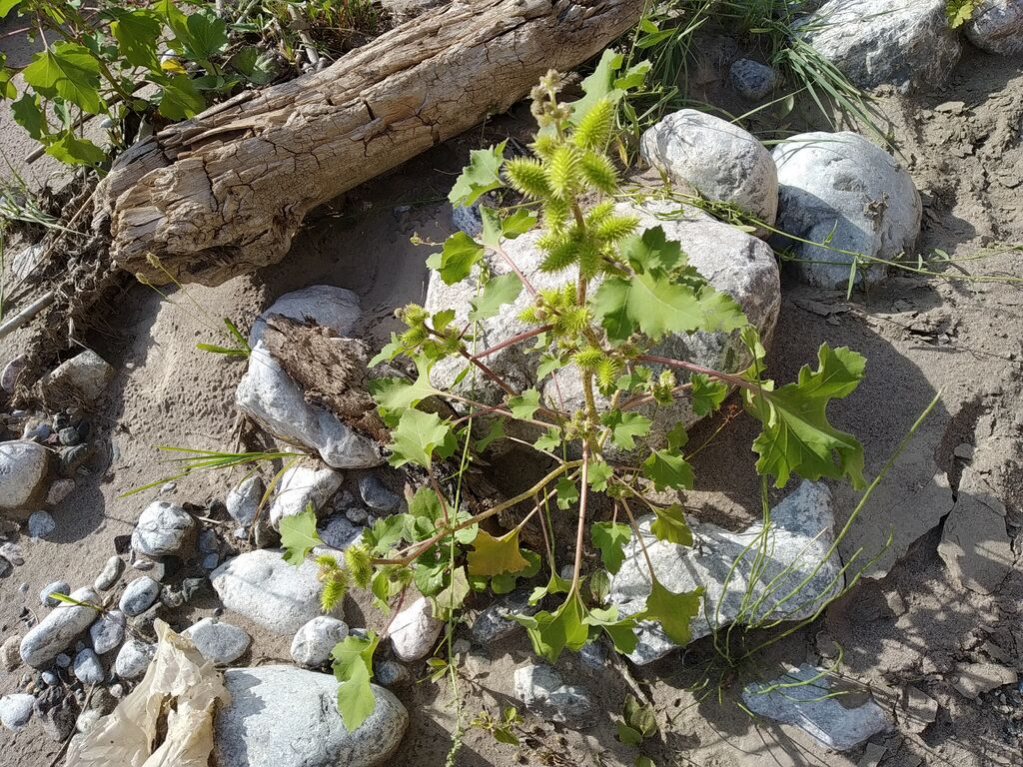
*Xanthiumorientale* along the Kyzyl-Suu River, Kyrgyzstan (photo by G. Lazkov, 19 August 2021).

According to herbarium collections, *X.orientale* occurs in a number of localities in or around the Chü, Ysyk-Köl and Fergana Depressions, climbing into the mountains as high as 2200 m and as far as 30 km from the depressions with their high levels of agricultural activity and human population density. Our field observations confirm its currently extensive dispersal (Fig. 9).

#### Ecology

Gravelly riversides in the native distribution area; disturbed grounds, gravelly roadsides, sandy and gravelly riversides, fallow fields in the secondary distribution area.

#### Biology

Annual.

#### Notes

The modern taxonomic concept in *Xanthium* and the correct name for this species were established by Greuter (2003) and Tomasello (2018). Our understanding of the diagnostic characters is based on our revision of historical collections and field observations and agrees with the treatment of Nabiev (1993).

The burrs in Central Asian plants are narrowly cylindrical, thus corresponding to X.orientalesubsp.californicum (Greene) Greuter (incl. *X.chinense* Mill.), as originally identified by Kinzikaeva (1988).

#### Introduction to Kyrgyzstan

##### Period of introduction

Neophyte.

The first collection from Kyrgyzstan (Suzak Town) is dated 1968. We assume that the species had arrived after the Second World War, in the 1960s, as a contaminant of wheat grain of North American origin.

##### Pathways of introduction

Transport - Contaminant: Seed contaminant.

Most likely, the species had arrived as a contaminant of wheat imported from Canada, in agreement with observations in Europe (e.g. Suominen 1979). In Australia, contamination of cotton seed and animal fur were reported as other main pathways of introduction (McMillan 1975).

Further dispersal occurred with domestic animals, water and transport.

##### Invasion status

Fully naturalised, highly invasive.

Judging from the tendencies in its distribution and expansion, *Xanthiumorientale* seems to be more adapted to the hot arid climate and may have a better prospect of naturalisation than its predecessor, *X.strumarium*.

##### Evidence of impact

Agriculture - major impact (abundant weed of fields, gardens and pastures, contamination of wool). Native ecosystems - major impact (local occurrence along mountain streams and roadsides in mountainous areas, forming extensive monodominant stands). Urban areas - major impact (ruderal occurrence, locally abundant).

##### Trend

Rapidly increasing (observed).

### Xanthium spinosum

L. 1753

BCC30C7A-F193-5B76-871D-F09D577CFCA8

urn:lsid:ipni.org:names:260892-1

Xanthium spinosum L., Sp. Pl. 2: 987 (1753) — *Acanthoxanthiumspinosum* (L.) Fourr., Ann. Soc. Linn. Lyon, sér. 2, 17: 110 (1869).

#### Diagnosis

This species is characterised by armed leaves and subglabrous elliptic burrs 1-1.5 cm long with numerous hooked prickles.

#### Distribution

##### Native distribution

The species is native to South America (Löve 1975).

##### Secondary distribution

Neophyte in North America, Europe (including the Mediterranean), Southern Africa, Asia, Australia. In Europe, this species belongs to the most widely distributed alien vascular plants (Lambdon et al. 2008).

##### Distribution in Central Asia

Widely distributed in all the countries (Nabiev 1993).

In Chinese Central Asia, *Xanthiumspinosum* was first recorded in the 1880s by A. Regel from Uqturpan County, Xinjiang Uygur Autonomous Region (Fedtschenko and Fedtschenko 1911). This early Chinese record has been neglected in national inventories (e.g. Xu et al. 2012).

In Kazakhstan, this species occurs in four restricted areas, of which the Talas Alatau is adjacent to Kyrgyzstan (Zaitseva 1965, Aldibekova et al. 2018). It was first observed in 1877 by I. Zarubin along the Syrdarya River between Qazaly (formerly Kazalinsk), Josaly (formerly Karmakshy) and Qyzylorda (formerly Perovsk) (Zarubin 1879), and then on the Maŋğystau Peninsula at the Kaspian Sea (first collected in 1895).

In Uzbekistan, the species has been originally known from the eastern parts of the country (Tashkent and Samarkand Regions) (Nabiev 1962b). The first observation made by A. Regel along the Salor irrigation channel near Tashkent was dated the 1880s (Fedtschenko and Fedtschenko 1911); the first specimens were collected in 1912-1920 near railway stations and along roadsides.

In Tajikistan, the species was first collected as a ruderal plant from Dushanbe (Grigoriev 1953), Xujand and Samgar in the northern part of the country (Komarov 1967). The date of the first record is not known, but seemingly it appeared shortly after the Second World War.

In Turkmenistan, the species occurred as a rare ruderal along irrrigation ditches in and around populated places (Nikitin 1960). The first herbarium specimen was collected in 1898.

As evident from the first herbarium specimens and observations in present-day Kazakhstan, the introduction of *X.spinosum* was linked to Russian fortifications that served as foreposts for the conquest and colonisation of the territory, and the roads connecting those fortifications along the Caspian Sea (established in the 1840s-1860s) and along the Syrdarya River (established in 1850s-1860s). As the species is notorious for its efficiency in contaminating various kinds of fur and wool (Kowarik and von der Lippe 2007), it is easy to understand that *X.spinosum* had arrived being tangled in manes and tails of Russian military horses, gradually proceeding eastwards (as, for example, in Australia: Woolls 1885). Since the species was found extensively established already in 1877 (Zarubin 1879), its invasion to Kazakhstan may have started in the 1860s or even earlier.

Its introduction to Turkmenistan was by the same military cavalry, probably in the 1880s. In particular, the first locality of *X.spinosum*, Daine Village (Dittrich 1989), was a border post which was certainly horse-served at that time. In Uzbekistan, the species appeared also in the 1880s, using the same pathway (Tashkent was the seat of the Russian administration in Turkestan, intensely supported by the military power from European Russia).

Besides the military traffic, by the 1850s, a road from Orsk Town along the Syr-Darya River was established for regular horse-driven transportation of merchandise from Russia to the Emirate of Buxoro and back (Nebolsin 1855), which undoubtedly promoted the further spread of *X.spinosum*.

The first records of the species from Kyrgyzstan are later and, therefore, are not linked with the horse power. Instead, they are firmly connected with the relocation of 2.3 million head of cattle during the second part of 1941, from the European part of the USSR to its Asiatic territories, including Central Asia (Kumanev 2006), as a contaminant of cattle tails and fur, fodder and bedding. The same pathway can be inferred also for Tajikistan, where the species was not registered before the War time.

##### Distribution in Kyrgyzstan

**Figure 10.**
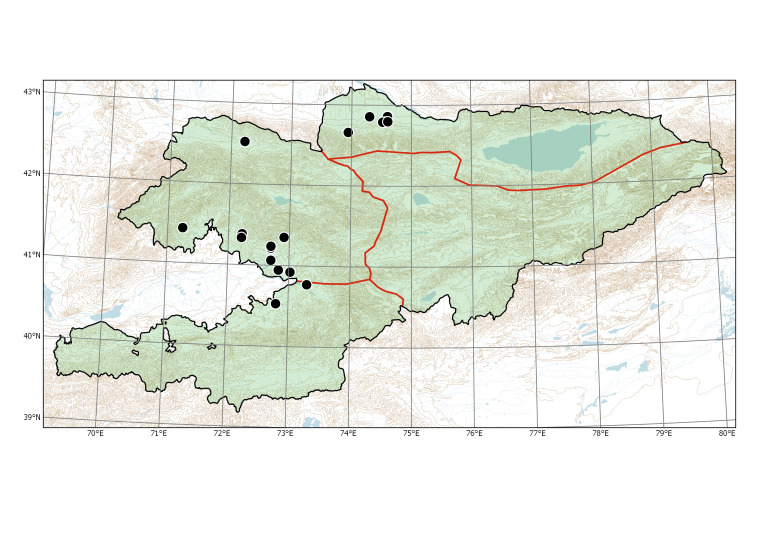
Distribution of *Xanthiumspinosum* in Kyrgyzstan according to herbarium specimens and our observations. The occurrence in Talas Region is borrowed from Deza (1983) and mapped tentatively.

Western Tian-Shan, Northern Tian-Shan, Alay-Turkestan (Fig. 10).

**Figure 11.**
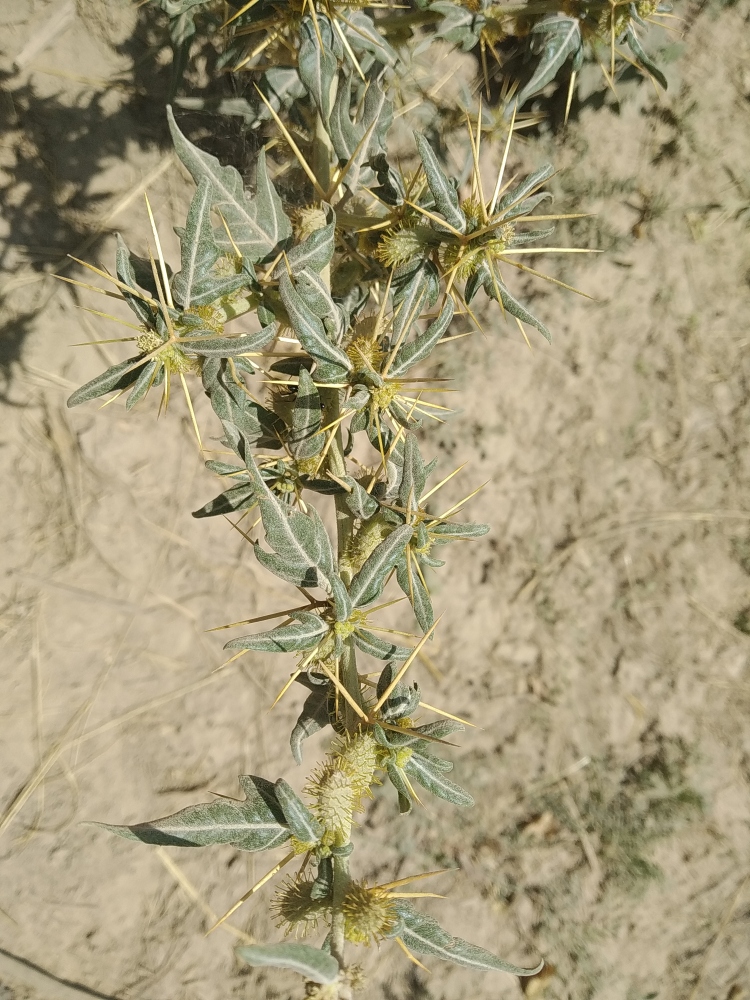
*Xanthiumspinosum* at the Toskool River, Kyrgyzstan (photo by G. Lazkov, 16 August 2021).

The species prefers arid areas with higher temperatures. It occurs in the Chü Depression with surrounding mountains and the eastern part of the Fergana Depression with surrounding mountains (Gorbunova 1965, Deza 1983); numerous recent observations exist (Fig. 11). Its first record comes from railway embankments in Bishkek City between Bishkek-1 Station (formerly Pishpek) and Bishkek-2 Station (formerly Frunze), where a large population was observed in 1942 (Nikitina 1965).

The species is also known from the Talas Depression in north-western Kyrgyzstan (Deza 1983), although without supporting specimens. This part of its distribution agrees with the corresponding occurrences in Kazakhstan (Aldibekova et al. 2018).

So far, the species was found in the lowlands and foothills, mostly at elevations of 600-1000 m, but also climbing up to 1650 m in the arid mountains.

#### Ecology

Riversides in the native distribution area; waste lands, disturbed grounds, roadsides, gravelly riversides, clayey lowlands, gardens and fallow fields in the secondary distribution area.

#### Biology

Annual.

#### Introduction to Kyrgyzstan

##### Period of introduction

Neophyte.

The first record from Kyrgyzstan is based on undocumented observations from railway tracks within Bishkek City, which are dated 1942 (Nikitina 1965). The first herbarium specimens were collected from Osh Town and dated 1946. Both records are connected with the Second World War migration of refugees and relocation of resources from the European part of the USSR during 1941, which required extensive transportation of industrial equipment, human population and livestock, including a massive amount of cattle and their supply (Kazakova and Salamov 1961, Kumanev 2006).

##### Pathways of introduction

Transport - Contaminant: Contaminant on animals.

The species has arrived as a contaminant on live animals, which were massively transported from south-eastern Europe. Further dispersal occurred by domestic animals and water.

In Europe, the species was also noted as a grain contaminant (e.g. Suominen 1979, Clement and Foster 1994).

##### Invasion status

Fully naturalised, invasive.

##### Evidence of impact

Agriculture - minor impact (the species may occur as a weed of vegetable plantations and was noticed in gardens and vineyards) (Deza 1983). Native ecosystems - major impact (recorded along mountain rivers, in mountain forests and in steppe-like vegetation around populated places, mostly along roadsides). Urban areas - major impact (recorded as a ruderal in many populated places).

##### Trend

Increasing (observed).

### Xanthium strumarium

L. 1753

60682FD4-F311-57B5-AB69-526935718333

urn:lsid:ipni.org:names:260893-1

Xanthium strumarium L., Sp. Pl. 2: 987 (1753).
Xanthium strumarium *Xanthiumchinense*
Xanthium strumarium *Xanthiumsibiricum*

#### Diagnosis

This species is characterised by unarmed leaves and broadly cylindrical burrs 1-2 cm long with less numerous, sparsely spaced hooked prickles.

#### Distribution

##### Native distribution

Pollen and macrofossil evidence suggests that the species is native to the southern temperate zone of Eurasia, stretching from Greece through the Black Sea Basin and the Caspian Sea Basin, including the Middle East and the Caucasus (Opravil 1983). Recent studies of palaeopalynological records indicate that the eastern limit of its native distribution may extend as far eastwards as to Central India, where its pollen was found in sediments dated over 12000 calibrated years before the present (Quamar and Kar 2020). The key area for the species seems to be Asia Minor and the South Caspian Region.

In Central Asia, *Xanthiumstrumarium* seems to be native in southern Turkmenistan (cf. Nikitin 1960).

##### Secondary distribution

The species is an archaeophyte in Europe (including a large part of the Mediterranean), Boreal, Central and probably Tropical Asia; it occurs as a neophyte in North and South America and Australia, where its distribution data may be obscured by the confusion with *X.orientale*.

Although the native distribution of *X.strumarium* was considered rather uncertain due to its early dispersal by human activities, its archaeophytic occurrence in Central Europe (Lange 1968, Opravil 1983, Sostarić et al. 2009) and a large part of the Mediterranean (Opravil 1983) was proven. In Europe, this species belongs to the most widely distributed alien vascular plants (Lambdon et al. 2008).

In north-western China (Xinjiang), according to the pollen data, the species was introduced about 3700 cal. years before the present, with the expansion of the Andronovo culture (Tarasov et al. 2019). The invasion of *X.strumarium* to Xinjiang corresponded to the introduction of wheat cultivation to the territory (Betts et al. 2014), and Iranian-speaking people of the Androsovo culture seem to have been responsible for both events.

Similarly, wheat cultivation was recorded as present some 4300-4000 cal. years before the present in the steppes near the Dzungarian Range, eastern Kazakhstan (Frachetti et al. 2010), likely indicating the corresponding expansion of *X.strumarium* across Central Asia.

The earliest archaeological data from Xinjiang, China (Sheng et al. 2018) recorded the presence and human use of the species 2200-2400 years ago; this record corresponds to the increase of the *Xanthium* pollen abundance linked with the intensified human activities through the Silk Road trade connections (Tarasov et al. 2019).

Wheat cultivation was introduced to Central Asia (Tajikistan and Uzbekistan) from Iran and Afghanistan ca. 5000 years ago (Spengler and Willcox 2013), and was most likely accompanied by the corresponding invasion of *X.strumarium*. The introduction of wheat was accompanied by the spread of sheep and taurine cattle in agro-pastoral cultures developed in piedmont areas along main mountain systems in Central Asia, on the way from Iran to north-western China (Spengler 2015, Stevens et al. 2016), which makes the probability for the arrival of *X.strumarium* even stronger because this species is a common contaminant of wool and is locally dispersed with sheep and cattle by exozoochory.

Preston et al. (2004) hypothesised that the commonly observed decline of archaeophytes, which occurred largely on arable lands and around human settlements, happened "perhaps because new introductions no longer balance the inevitable losses". We think this explanation is highly likely in the case of *X.strumarium*. Although this species has a clear adaptation to zoochory and is frequently quoted in connection with this fact, in Kyrgyzstan it was most common on fields and in populated places rather than on pastures (Deza 1983), thus indicating that the main vector of its local dispersal was management of arable lands. This management implied a constant arrival of new diaspores through contaminated seed. After the Russian colonisation of Central Asia, the original wheat cultivars were replaced by foreign selections due to the constantly increasing demand for higher yields, and the seed material became imported from remote territories. This change implied that the local circulation of wheat weed seeds had stopped or was at least obstructed.

At some point, source fields of the imported wheat seed became infested by *X.orientale* rather than *X.strumarium* (cf. Suominen 1979), and new diaspores of *X.strumarium* no longer arrived to the Central Asian fields. The lack of the outsource renewal, coupled with the constant management of fields and a limited extent of full naturalisation in native habitats, had likely caused a prominent decline of *X.strumarium* in Central Asia. A similar process had occurred at the same time period in Boreal Europe (e.g. Gudžinskas 1997). In Central Europe, *X.spinosum* had experienced a similar decline with the recent advancement of agriculture and wool cleaning (e.g. Dudáš and Eliáš Jr. 2021).

According to herbarium collections, in Central Asia, *X.strumarium* was most likely naturalised in steppe areas along rivers, especially in Kazakhstan. In Kyrgyzstan, its naturalised populations were probably concentrated in the north, along the Chü River, which should be explored for relic occurrences of this species; this territory was found climatically suitable for the naturalisation of *X.strumarium* s.l., based on the data derived from the current invasion of *X.orientale* (Zhang et al. 2021). In other territories, its former occurrences around fields and populated places seem to have been largely ephemeral.

**Figure 12.**
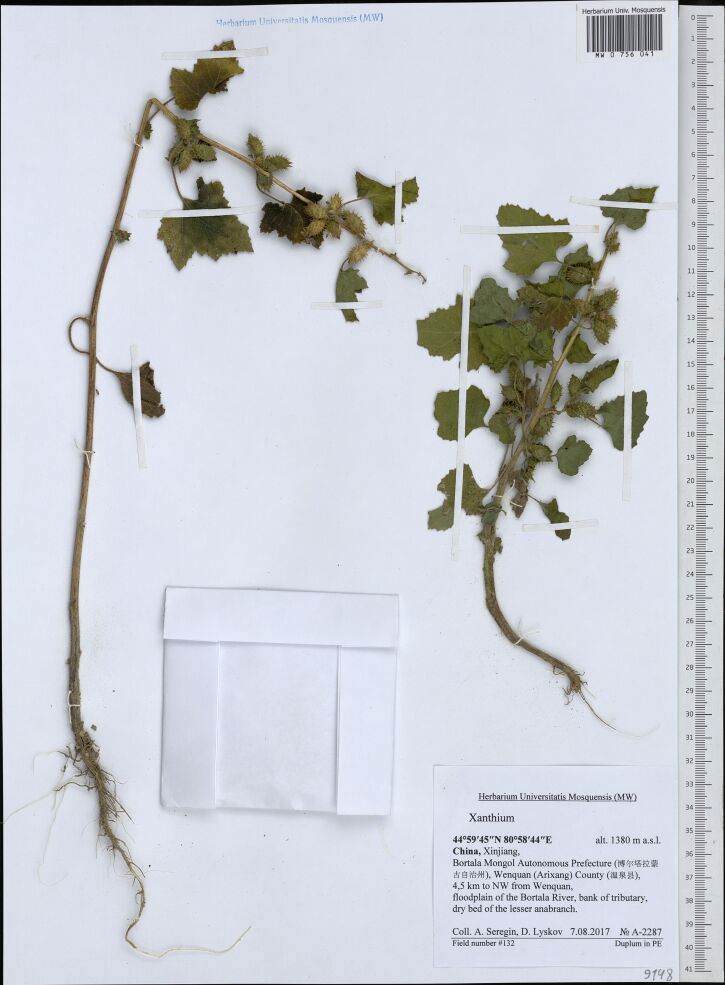
A recent specimen of *Xanthiumstrumarium* from northern Xinjiang, China (MW075041).

The latest records of *X.strumarium* are few, and a special effort is required to trace its refugia. We are not aware of any recent collection or observation from Central Asia, whereas one recent specimen was traced from agricultural valleys of northern Xinjiang, China (Fig. 12; Seregin 2021).

##### Distribution in Central Asia

The species was present in all the countries of Central Asia (Nabiev 1993).

##### Distribution in Kyrgyzstan

**Figure 13.**
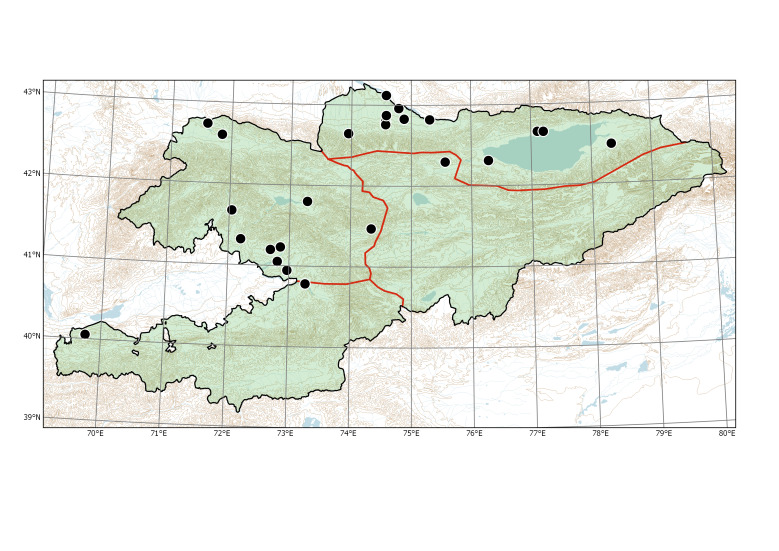
Historical distribution of *Xanthiumstrumarium* in Kyrgyzstan according to the specimens examined.

Western Tian-Shan, Northern Tian-Shan, Eastern Tian-Shan, Alay-Turkestan (Fig. 13).

The species has been considered occurring in all parts of the country (Gorbunova 1965, Deza 1983) and was collected from all phytogeographic regions. It occurred in or along all major depressions and valleys.

According to herbarium specimens, the species was found at elevations between 650 and 2100 m, thus covering the territories suitable for agriculture.

#### Ecology

Same as for *Xanthiumorientale*, but probably less competitive and more confined to steppes rather than to arid depressions; in Central Asia, *X.strumarium* may reach the altitudes as high as 4000 m (Nabiev 1993). In Kyrgyzstan, both species occurred in the same habitats.

#### Biology

Annual.

#### Introduction to Kyrgyzstan

##### Period of introduction

Archaeophyte.

The species is an archaeophyte of the Neolithic period, which had arrived with a further development of agriculture.

##### Pathways of introduction

Transport - Contaminant: Seed contaminant.

The species had likely arrived with the cultivation of wheat, which was introduced to present-day Tajikistan and Uzbekistan no later than 5000 years ago (Spengler and Willcox 2013). Further dispersal occurred by domestic animals and water (Ridley 1930).

##### Invasion status

Naturalised, not invasive. Historically common and abundant, but likely dependent on human management; currently nearly extinct, but probably still resident in the country (current presence is not confirmed, last record dated 1978).

##### Evidence of impact

Agriculture - minor impact (formerly common weed of fields, gardens and pastures, contaminant of wool; currently not recorded). Native ecosystems - minor impact (formerly extensive occurrence along mountain streams and in steppe-like landscapes around populated places; present-day occurrence is not confirmed). Urban areas - minor impact (former ruderal occurrence).

##### Trend

Strongly declining (observed).

## Discussion

The non-native plant species discussed in the present contribution had arrived to Kyrgyzstan in various times and by various means.

The arrival of a few species was associated with a certain event of human migrations (*Xanthiumspinosum*: European refugees of the Second World War) or agricultural development (*Xanthiumstrumarium*: adoption of wheat cultivation and sheep keeping in the Neolithic period), many other arrivals were associated with the recent import of grain and forage (*Buniasorientalis* and *Erigeronannuus*: imported forage in the period of independence; *Xanthiumorientale*: imported grain in the 1960s; *Bidenstinctoria* and *Buniasorientalis*: imported grain in the period of independence), whereas one more immigrant was a weed of ornamental plants (*Erigeronlilacinus*: introduced with ornamental plants via nurseries in the 2010s).

All the species have arrived as contaminants of various kinds. All these species are known as weedy in various parts of their distribution areas and remain such also in Kyrgyzstan, with the exception of *Bidenstinctoria* which is not established in the territory. The three species of *Xanthium* have become highly invasive in the territory, whereas *Erigeronannuus* is invasive but has not expanded to a large area yet. *Buniasorientalis* and *Erigeronlilacinus* are locally established but potentially invasive and may move to that category with time. Lastly, we do not expect that *Bidenstinctoria* may become established in Kyrgyzstan.

The origin of these alien plants is mostly the New World (*Bidenstinctoria*, *Erigeronannuus*, *E.lilacinus*, *Xanthiumorientale*, *X.spinosum*), whereas two species were introduced from Eastern Europe (*Buniasorientalis*) or southern Temperate Asia (*Xanthiumstrumarium*).

The strong decline of *Xanthumstrumarium*, which was among the oldest archaeophytes and noxious weeds in Kyrgyzstan, suggests that even old invasive plants may share the fate of other crop weeds, if they are largely confined to arable lands and rely on crop management. When the drivers sustaining such plants cease to operate, such formerly invasive species may appear not to fit the local climatic environment and natural dispersal agents, thus experiencing a strong decline or even extinction. This conclusion corroborates the superficially paradoxical outcomes of the study by Thomas and Palmer (2015), who concluded that the long national lists of invasive plants *per se* do not indicate a strong deterioration of natural environments and threats to the native flora; other factors (human-independent pathways of dispersal, species-environment interactions) should be considered in the evaluation of harmful effects of individual alien plant species and the scale of their invasion to the native ecosystems outside the man-made and man-sustained environments.

In the complex history of *Xanthiumspinosum* in Central Asia, only the second wave of its invasion has reached Kyrgyzstan. This species is a special case of polemochores (war-time immigrants), i.e. alien plants introduced in connection with military activities that caused long-distance migrations of human population and extensive transportation of their supply (e.g. Sennikov 2012).

Another special case among these introductions is *Erigeronlilacinus*, in which the national Botanical Garden has been partly involved. Although botanical gardens are considered as a major proven source of global plant invasions (Hulme 2011), the scale of this particular introduction seems to have been very limited but, at the same time, the naturalisation success in the Botanical Garden was certainly far the greatest. This case justifies warnings of other researchers (e.g. Mayorov et al. 2021) that the level of biocontrol should be further increased in botanical gardens (Hulme 2011, Wondafrash et al. 2021).

## Supplementary Material

XML Treatment for Bidens tinctoria

XML Treatment for Bunias orientalis

XML Treatment for Erigeron annuus

XML Treatment for Erigeron lilacinus

XML Treatment for Xanthium orientale

XML Treatment for Xanthium spinosum

XML Treatment for Xanthium strumarium

2572E556-1DCA-57E9-AC04-A79D21A2C8E5 10.3897/BDJ.9.e75590.suppl1 Supplementary material 1 Occurrence records of alien plants from Kyrgyzstan (contribution 1) Data type occurrences Brief description This is a comprehensive dataset of all occurrences of non-native vascular plants in Kyrgyzstan (contribution 1). The dataset is based on herbarium specimens, documented observations of the authors and other people, observations traced from reliable literature, and undocumented observations of the authors. The dataset contains 113 records and is complete as of 22 October 2021. File: oo_602436.xlsx https://binary.pensoft.net/file/602436 Sennikov, Alexander; Lazkov, Georgy
